# Health-related quality of life among adults living with chronic non-communicable diseases in the Ho Municipality of Ghana: a health facility-based cross-sectional study

**DOI:** 10.1186/s12889-024-18143-3

**Published:** 2024-03-06

**Authors:** William Kwame Witts, Hubert Amu, Robert Kokou Dowou, Frank Oppong Kwafo, Luchuo Engelbert Bain

**Affiliations:** 1 Department of Epidemiology and Biostatistics, F.N. Binka School of Public Health, University of Health and Allied Sciences, Hohoe, Ghana; 2 Department of Population and Behavioural Sciences, F.N. Binka School of Public Health, University of Health and Allied Sciences, Hohoe, Ghana; 3 Department of Psychology, Faculty of Humanities, University of Johannesburg, Johannesburg, Auckland Park, South Africa; 4 International Development Research Centre, IDRC, Ottawa, Canada

**Keywords:** Health-related quality of life (HRQoL), Chronic non-communicable diseases (CNCDs), Ghana, Ho Municipality

## Abstract

**Background:**

Morbidity and mortality rates from chronic non-communicable diseases (CNCDs) are increasing globally. In Ghana, CNCDs account for 43% of all deaths. We examined the Health-Related Quality of Life (HRQoL) and associated factors among adults living with CNCDs in the Ho Municipality.

**Methods:**

This was a health facility-based descriptive cross-sectional study among 432 adults living with cancer, diabetes, chronic kidney disease (CKD), stroke, and hypertension in the Ho Municipality of Ghana. The study adopted the EQ-5D-5L instrument and the Ugandan value set to compute respondents’ HRQoL index. Quantile regression models were used in analysing the data with STATA v17.0 at 95% Confidence Intervals, and statistical significance set at *p* < 0.05.

**Results:**

63.7% of our respondents reported having a problem across the five dimensions of the EQ-5D-5L. The most problems were reported in the dimensions “Anxiety/Depression” (94.4%) and “Pain/Discomfort” (91.4%). Divorced/separated respondents (aOR=-0.52, 95% CI=-0.71, -0.33) and those living with comorbidities (aOR=-0.95, 95% CI=-0.15, -0.04,) were less likely to report high index for HRQoL. However, respondents diagnosed with CKD (aOR = 0.26, 95% CI = 0.10, 0.42), diabetes (aOR = 0.28, 95% CI = 0.11, 0.45), hypertension (aOR = 0.35, 95% CI = 0.19, 0.50) and stroke (aOR = 0.26, 95% CI = 0.11, 0.40) were more likely to report higher index than those diagnosed with cancer.

**Conclusion:**

Our study revealed elevated proportions of reported problems in the “Anxiety/Depression” and “Pain/Discomfort” dimensions, indicating noteworthy concerns in these areas of HRQoL. The prevalent issues reported across HRQoL dimensions are cause for concern, posing potential exacerbation of health conditions. We advocate for collaborative efforts from the Ministry of Health, Ghana Health Service, and relevant stakeholders to scrutinize and implement interventions targeting social and psychological factors. These efforts should specifically address contributors to diminished health-related quality of life, particularly among less educated, divorced, and comorbid individuals.

**Supplementary Information:**

The online version contains supplementary material available at 10.1186/s12889-024-18143-3.

## Background

Several global interventions to improve health outcomes have been implemented over the last two decades. The Sustainable Development Goals (SDGs) promulgated in 2015, for example, seek to ensure that developing countries around the world can achieve 17 development-oriented goals by 2030 [1]. Goal three aims to ensure healthy lives and promote well-being for all people of all ages, and target 3.4 specifically aims to reduce premature mortality from noncommunicable diseases by one-third by 2030 [1].

Despite efforts by various countries to meet the SDG targets, chronic illness remains a major global health issue, with the number of people affected steadily increasing [2]. About 41 million people worldwide die of CNCDs each year. This represents 71% of all deaths. Most of those deaths were caused by just four CNCDs: cardiovascular diseases (17.9 million deaths), cancer (9 million deaths), chronic respiratory diseases (3.8 million deaths), and diabetes (1.6 million deaths) [3]. With the obvious chronic nature of these non-communicable diseases, it is evident that CNCDs have become the heaviest burden to healthcare systems globally [4, 5].

CNCDs are the main causes of death in low- and middle-income countries (LMIC) [6], and they come at a high cost that goes beyond health, trapping people in poverty, denying them a dignified life, undermining labour productivity, and jeopardising economic growth [7]. CNCDs are also becoming a problem via widening gaps in opportunity, wealth, and power. Their effect on low- and lower-middle-income countries is a significant barrier. In all nations, the poorest and most vulnerable communities are the most at risk and have the least access to the services needed to detect and treat CNCDs [8].

Over the previous two decades, disability and death rates from chronic diseases such as diabetes, hypertension, and stroke have increased in many Sub-Saharan Africa (SSA) countries [9, 10]. In the same manner, Ghana’s position is no different. The WHO reported that CNCDs account for around 43% of all deaths in Ghana. Cardiovascular illnesses represented 19%; malignancies, 5%; chronic respiratory diseases, 3%; and other CNCDs, 13%. Ghana similarly reported a 21% risk of premature mortality due to CNCDs [8].

These records inherently support the interest in chronically ill patients’ health-related quality of life (HRQoL) as a key health outcome indicator, with a growing body of literature over the past few years [11]. This is because of the absence of an adequate cure for several CNCDs [12]. Empirical evidence has proven that there has been a significant decrease in HRQoL in patients with CNCDs and indicated HRQoL as an important predictor of morbidity and mortality [13, 14].

Findings from a study conducted in three European countries (Italy, Spain, and Greece) revealed that the quality of life (QoL) worsens for physically inactive people and those with multiple CNCDs. The same study also posited that there is a direct association between QoL and health perception [15].

On the other hand, a Chinese study that sought to measure the QoL among hypertensive patients found the perceived economic burden caused by hypertension to be the most common factor impacting the patient’s HRQoL. They also found that females were more susceptible to suffering from a decreased QoL as a result [16] An Ethiopian study that measured the HRQoL among diabetic patients found a high overall HRQoL score with social domains having a higher mean score. It also found a statistically significant association between educational status, marital status, occupation, duration of diabetes as well as diabetes-related complications and HRQoL [17]. Another study by Abegaz et al. [18] in Ethiopia which measured HRQoL among cancer patients revealed that HRQoL of cancer patients in Ethiopia is comparatively low. The study cited the unmet needs of cancer patients and the level of satisfaction with the overall care to be influencing the extent of HRQoL. The previous literature has shown that numerous elements might impact an individual’s HRQoL while suffering from CNCDs, yet HRQoL can also be an independent factor that impacts other areas of an individual’s life. Although there is a growing number of pieces of literature that seek to investigate the HRQoL of individuals, there remains a paucity in the literature on the HRQoL of persons living with CNCDs. Hence, this study sought to investigate the HRQoL of adults living with CNCDs and the factors influencing it. The findings could positively inform policy decisions on the management of CNCDs in Ghana and beyond.

## Theoretical issues

This study was underpinned by the Social Ecological Model (SEM), which was originally conceptualised to understand human development. Urie Bronfenbrenner, the main proponent of the SEM introduced this modelintroduced this model in 1979. Subsequently, numerous scholarly inquiries have embraced and adapted this model to delineate the diverse strata of influence within a specific milieu [19]. The SEM posits that individuals are subject to multifaceted influences at five distinct societal levels, thereby elucidating the complex interplay of factors shaping human behaviour and development within a given context.

In his initial theory, Bronfenbrenner postulated that the entire ecological system in which growth occurs needs to be considered to understand human development. In subsequent revisions, Bronfenbrenner acknowledged the relevance of biological and genetic aspects of the person in human development [20].

The ecological approach provides a useful framework for comprehending the various aspects that influence health and well-being. It is a model that can help provide a comprehensive view of the elements that influence certain health behaviours, including social determinants of health. The multiple levels of influence recognized under the social-ecological models include intrapersonal/individual factors, interpersonal factors, institutional or organizational factors, community factors and public policy factors.

### Potential factors to influence health-related quality of life

**Fig. 1:**
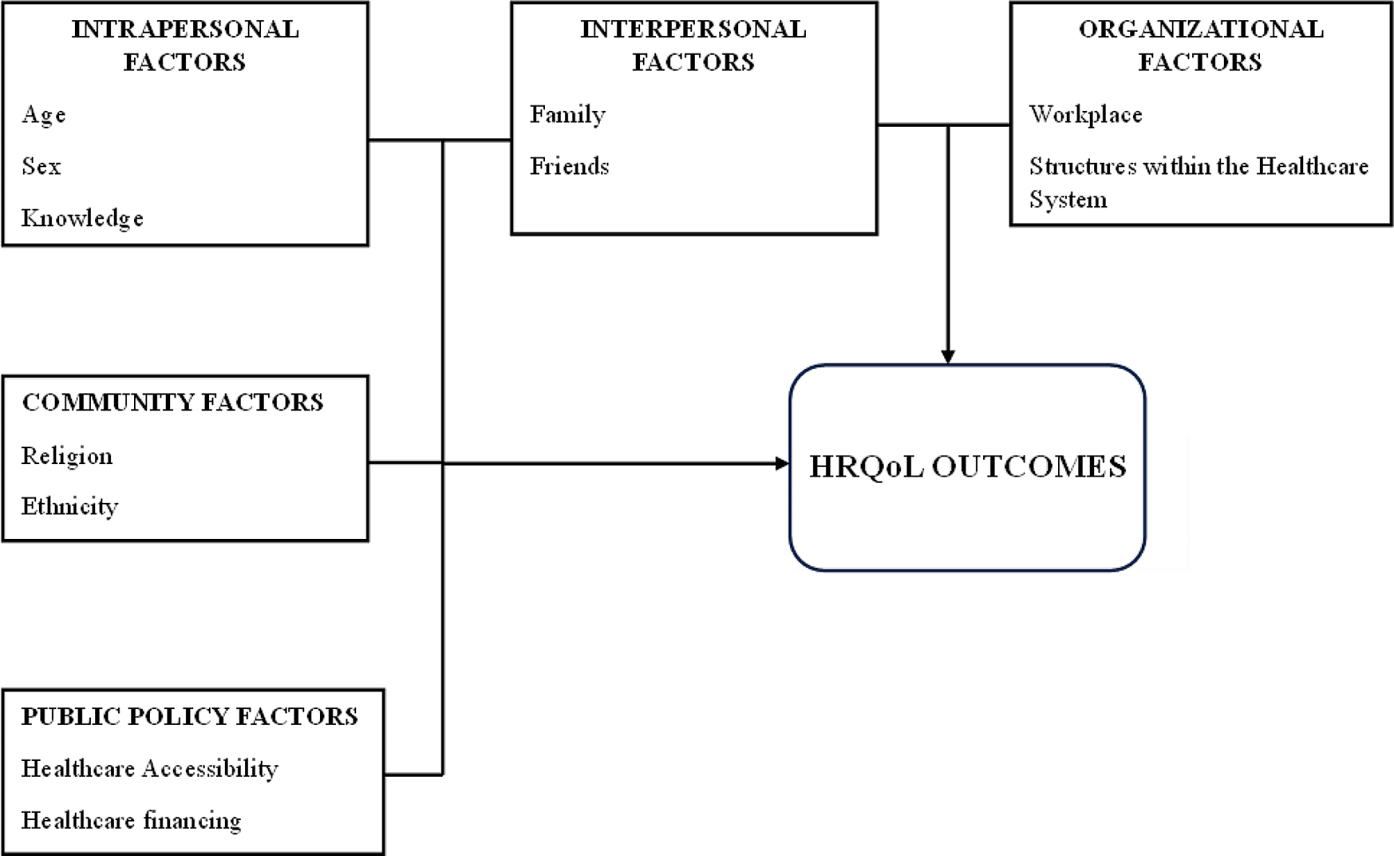
Conceptual framework

In this study, intrapersonal factors, including specific intrinsic socio-demographic variables such as age, sex, and educational level, were systematically examined to gauge their predictive capacity concerning Health-Related Quality of Life (HRQoL) [21, 22]. Concurrently, interpersonal and community-level variables such as marital status, religious affiliations, and ethnic background were scrutinized. These variables were of particular interest due to their roles as conduits for social support, a factor posited to significantly impact HRQoL [23]. Additionally, the study delved into individuals’ diagnoses of select Chronic Non-Communicable Diseases (CNCDs), the duration of their conditions, and any comorbidities they might be managing. This comprehensive investigation was motivated by the understanding that these variables are likely influenced by one or more domains at various levels within the Social Ecological Model (SEM). To visually represent the intricate interplay of these factors, we developed Fig. 1, a conceptual framework aligned with the a priori hypotheses guiding our study.

## Materials and methods

### Study site

The Ho Teaching Hospital (HTH), situated in the Ho Municipality of the Volta Region, is one of the latest teaching hospitals in Ghana. The hospital provides both out-patient and in-patient services where walk-in patients can receive general or specialized consultations, and prolonged treatment and observation respectively. The hospital has five clinical departments: internal medicine, surgery, obstetrics and gynaecology, child health, and public health. The HTH’s clinical services are broadly divided into two categories: general and specialist clinical services. General services include general surgery, urology, paediatrics, orthopaedics, etc., whereas specialist services are delivered by disease-/condition-specific clinics such as the diabetes clinic, fertility clinic, physiotherapy, anti-retroviral therapy clinic, and eye clinic.

The HTH has been chosen for this study because it has become one of the top hospitals in the country and receives both primary and referral cases from the entire Volta region and parts of the neighbouring regions in Ghana.

### Data source and study design

This was a health facility-based study that utilized a quantitative descriptive cross-sectional design. Data obtained from respondents was used to determine the HRQoL of adults living with chronic non-communicable diseases. The primary target population of this study was adults diagnosed with hypertension, cancer (breast, cervical, prostate, or lung cancers), chronic kidney disease (CKD), stroke, or diabetes. Adults with any of the CNCDs of interest who visited the Ho Teaching Hospital’s disease-/condition-specific clinics, as well as chronic disease patients in in-patient care units, were included in the study. In contrast, adults with any of the CNCDs of interest who visited the hospital’s disease-/condition-specific clinics but were foreign nationals, severely or terminally ill and unable to effectively communicate were excluded from participating in this study. A probabilistic sampling method utilizing the simple random sampling technique was adopted in recruiting respondents for the study. We relied on the Strengthening the Reporting of Observational Studies in Epidemiology (STROBE) statement in writing the manuscript [see Additional File 1] [24].

### Sample size determination

In this study, a total of 432 adults living with CNCDs were included in the final analysis. This sample size was determined using the single proportion population formula by Cochran (1977): n= [z² p (1-p)]/d². The sample size was estimated based on 50% (0.5) prevalence estimates of CNCDs in Ghana as used by Osei et al. in their 2021 study [25].

### Data collection and instrument

Structured interviewer-administered questionnaires were used in collecting data from respondents who met the eligibility criteria. Even though the data were collected at the hospital, the interviews were conducted at locations where others could not listen to the responses to the questions. The data collection instrument utilized consisted of two sections with the first section gathering respondents’ socio-demographic characteristics and the second section gathering data on respondents’ HRQoL [see Additional File 2]. The study adopted the EQ-5D-5L protocol from EuroQol to measure the HRQoL of patients. The questionnaire was mainly administered in English although a proportion was administered in the Ghanaian language (Ewe) local to the study setting. To ensure that the use of the local language did not affect the quality of data collected, the training organised for assistants also focused on interpretations of the instrument in the language to ensure that the right questions were asked. Before the main data collection, the instrument was pre-tested among 50 CNCD patients in the Volta Regional Hospital in the Volta Region of Ghana. The pre-testing helped to fine-tune the choices of words to use in the interpretation of the instrument in the local language.

## Study variables

### Outcome variable

HRQoL was the main outcome variable in this study. This was measured using the EQ-5D-5L protocol from EuroQol. The protocol measured five different dimensions of an individual’s health to posit the overall HRQoL. These dimensions included “Mobility”, “Self-care”, “Usual Activities”, “Pain/Discomfort”, and “Anxiety/Depression”. Each dimension, hence, had five levels of responses which measured respondents’ state or functionality across the dimensions. The responses according to the levels were “No problem”, “Slight problem”, “Moderate problem”, “Severe problem”, and “Extreme problem/Unable to do”. The validity and reliability of the EQ-5D-5L protocol are adequately established in other studies [26–29].

### Explanatory variables

Age, sex, marital status, education, religion, ethnicity, diagnosed CNCD, diagnosis duration, comorbidity status, specific comorbidities, recommended behavioural/lifestyle changes: physical activity, dietary changes, smoke cessation, and alcohol intake moderation were the explanatory variables used for this study [see Additional File 3]. These variables were selected based on their relevance to the study and as found by other studies [25, 30].

### Statistical analyses

Data analyses were done using Stata software version 17.0 (Stata Corporation, College Station, TX, USA). We adopted the Ugandan EQ-5D-5 L value set to compute the HRQoL index for this study [31]. Descriptive summary statistics were estimated for socio-demographic variables and EQ-5D-5L dimensions. As the EQ-5D-5L index was non-normally distributed, the differences between the index and explanatory variables were computed using the median, lower and upper quartiles. A Kruskal-Wallis test was performed to establish statistical significance between explanatory variables and the outcome variable. A quantile regression was performed to estimate the effect of the explanatory variables on the HRQoL index of respondents. A p-value of 0.05 or less was considered statistically significant in this study. Variables that showed significance (*p* < 0.05) in the bivariate quantile regression analysis were included in the multivariable quantile regression model in which statistical significance was considered at *p* < 0.05.

## Results

### Socio-demographic characteristics of the patients

Table 1 presents the background characteristics of thepatients. The mean age of patients was 59 years with a standard deviation of 11.31. About 48% were 60 years and above, and 52.5%, were females. Additionally, 69.7% of the study patients were married whereas 40.7% had a tertiary-level education. A total of 83.3% reported being Christians and 54.4% reported being Ewes. Amongst the five CNCDs of interest in this study, 43.5% of the total study population reported being diagnosed with hypertension. However, the results showed that 40.7% of the study respondents also reported living with the various conditions for a minimum of a year and a maximum of five years while only 38.7% of the study population reported living with at least one comorbidity. Furthermore, 99.1% of the study respondents reported being on behavioural and lifestyle treatment with 84.7%, 92.7%, and 16.5% respondents reporting being recommended dietary changes, physical exercise/activity, and alcohol intake cessation, respectively.

**Table 1:** Socio-demographic characteristics of respondents

Variables	Frequency [*N* = 432]	Percentage [%]
**Age [Mean (SD)]**	**[59 (11.31)]**	
30–39	38	8.8
40–49	43	9.9
50–59	145	33.6
60+	206	47.7
**Sex**		
Male	205	47.5
Female	227	52.5
**Marital Status**		
Never married	34	7.9
Married	301	69.7
Divorced/Separated	20	4.6
Widowed	77	17.8
**Highest Education Level**		
No formal education	16	3.7
Primary	28	6.5
JHS/JSS/Middle School	100	23.2
SHS/SSS/O-Level	112	25.9
Tertiary	176	40.7
**Religion**		
Christianity	360	83.3
Islam	67	15.5
African Traditional	5	1.2
**Ethnicity**		
Akan	140	32.4
Ewe	235	54.4
Guan	30	6.9
Ga/Dangme	27	6.3
**Diagnosed CNCD**		
Cancer	16	3.7
Chronic Kidney Disease	56	13.0
Diabetes	52	12.0
Hypertension	188	43.5
Stroke	120	27.8
**Cancer Type**		
Breast Cancer	6	37.50
Prostate Cancer	9	56.3
Bladder Cancer	1	6.2
**Diagnosis Duration (**Year)		
< 1	97	22.5
1–5	176	40.7
6–10	101	23.4
10+	58	13.4
**Living With Comorbidities**		
Yes	167	38.7
No	265	61.3
**Comorbidities**		
Arthritis	4	2.45
Bodily pains	4	2.45
Diabetes	20	12.3
Diabetes &Hypertension	8	4.9
Hypertension	110	67.5
Hernia	1	0.6
Peptic ulcer	4	2.45
Rheumatism	4	2.45
Stroke	8	4.9
**Are you currently on any behavioural/lifestyle treatment?**		
No	4	0.9
Yes	428	99.1
**Recommended behavioural/lifestyle changes (n = 428)**		
**Dietary Changes**		
No	65	15.3
Yes	359	84.7
**Physical Activity**		
No	31	7.3
Yes	393	92.7
**Smoke Cessation**		
No	422	99.5
Yes	2	0.5
**Alcohol Intake Moderation**		
No	354	83.5
Yes	70	16.5
**How often do you adhere to these recommendation(s)?**		
All the time	117	27.3
Most of the time	245	57.3
Sometimes	54	12.6
Rarely	12	2.8
**How often do you go for your follow-up/check-up / review?**		
Weekly	133	31.1
Every two weeks	73	17.1
Monthly	141	32.9
Every two months	68	15.9
Every three months	13	3.0
**How often do you adhere to your scheduled appointments for your follow-up/check-up / review?**		
All the time	184	43.0
Most of the time	239	55.8
Sometimes	0	0
Rarely	5	1.2

### Summary statistics of respondents’ HRQoL index using median, lower and upper quartiles

Table 2 below provides the summary statistics of the five dimensions of the EQ-5D-5 L utilizing the medians of each dimension as well as the lower and upper quartiles to illustrate the distribution of the respondents’ health-related quality of life index.

**Table 2:** Summary statistics of the EQ-5D-5 L using median, lower and upper quartiles

Dimensions	Median	P25	P75
Mobility	0.073	0	0.146
Self-care	0.068	0	0.068
Usual Activities	0.06	0	0.081
Pain/Discomfort	0.082	0.082	0.138
Anxiety/Depression	0.05	0.05	0.127

### Distribution of levels of perceived problems based on EQ-5D-5L dimension

Table 3 presents the distribution of levels of the perceived problem by respondents based on the dimensions of the EQ-5D-5L. It was found that the majority of respondents reported having “slight problem” and “moderate problem” across the five dimensions. The “anxiety/depression” and “usual activities” saw 2.3% and 1.8% of respondents reporting extreme problems.

**Table 3:** Distribution across EQ-5D-5L dimensions

	Mobility n (%)	Self-Care n (%)	Usual Activities n (%)	Pain/Discomfort n (%)	Anxiety/Depression n (%)
Level 1 **(**No Problem)	113 (26.2)	174(40.3)	117(27.1)	37(8.6)	24(5.6)
Level 2 **(**Slight Problem)	169(39.1)	176(40.7)	149(34.5)	185 (42.8)	215 (49.8)
Level 3 (Moderate Problem)	124(28.7)	70(16.2)	130(30.1)	150(34.7)	172(39.8)
Level 4 (Severe Problem)	21(4.8)	8(1.9)	28(6.5)	60(13.9)	11(2.5)
Level 5 (Extreme Problem / Unable to do)	5(1.2)	4(0.9)	8(1.8)		10(2.3)
Total	**432(100.00)**	**432(100.00)**	**432(100.00)**	**432(100.00)**	**432(100.00)**

Table 4 shows the distribution of perceived problem levels across the EQ-5D-5L dimensions based on two levels of problem categorization: “No problem” and “Some problem.” More than half of the respondents reported having a problem in at least one of the five dimensions. The most problems were reported in the dimensions “Anxiety/Depression” (94.4%) and “Pain/Discomfort” (91.4%).

**Table 4:** Distribution of levels of perceived problems using two levels of categorization

Dimensions	No Problem n (%)	Some Problem n (%)
Mobility	113 (26.2)	319 (73.8)
Self-care	174 (40.3)	258 (59.7)
Usual Activities	117 (27.1)	315 (72.9)
Pain/Discomfort	37 (9.0)	395 (91.4)
Anxiety/Depression	24 (5.6)	408 (94.4)

**Fig. 2:**
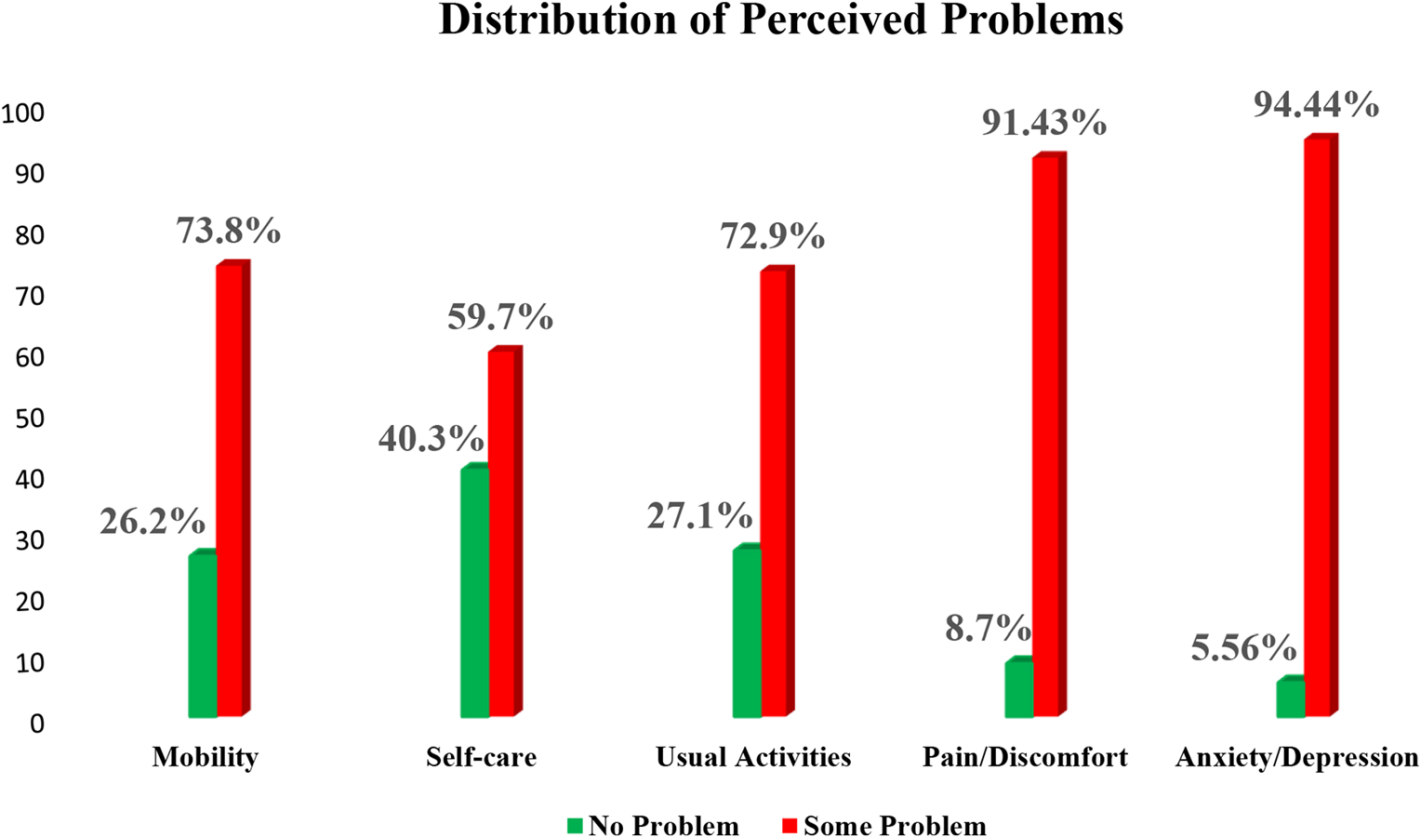
Distribution of perceived problems according to dimensions

Figure 2 shows the distribution of perceived problem levels across the EQ-5D-5L dimensions based on two levels of problem categorization: “No problem” and “Some problem”, as recommended by the EuroQol Research Foundation [23]. More than half of the respondents reported having a problem in at least one of the five dimensions. The most problems were reported in the dimensions “Anxiety/Depression” (94.4%) and “Pain/Discomfort” (91.4%).

### Predictors of HRQoL

#### Quantile regression of health-related quality of life

Table 5 presents the quantile regression analysis performed on respondents’ background characteristics and HRQoL. The results show that respondents who were divorced/separated (aOR=-0.52, 95% CI=-0.71, -0.33, p = < 0.001) were less likely to report a high index for HRQoL. However, our result posited that respondents diagnosed with CKD (aOR = 0.26, 95% CI = 0.10, 0.42, *p* = 0.001), diabetes (aOR = 0.28, 95% CI = 0.11, 0.45, *p* = 0.001), hypertension (aOR = 0.35, 95% CI = 0.19, 0.50, p = < 0.001) and stroke (aOR = 0.26, 95% CI = 0.11, 0.40, *p* = 0.001) were all more likely to report a high index for HRQoL compared to respondents diagnosed with cancer but respondents who reported living with other comorbidities (aOR =-0.95, 95% CI=-0.15, -0.04, *p* = 0.001) were less likely to report a high index for HRQoL. Additionally, we found that respondents who reported being recommended a change in dietary habits (aOR = 0.22, 95% CI = 0.13, 0.31, p = < 0.001) were more likely to report a high index for HRQoL.

**Table 5:** Quantile regression model on the predictors of health-related quality of life

Variables	P50 (P25, P75)	p-value	HRQoL Index			
			cOR	95% CI	aOR	95% CI
**Age**		**0.0001**				
30–39	0.868 (0.667, 0.868)		Ref		Ref	
40–49	0.765 (0.662, 0868)		-0.103	-0.24, 0.04	0.6	-0.09, 0.20
50–59	0.636 (0.496, 0.718)		-0.232	-0.35, -0.12***	0.02	-0.12, 0.17
60+	0.517 (0.268, 0.667)		-0.351	-0.46, -0.24***	-0.06	-0.21, 0.09
**Sex**		**0.0414**				
Female	0.538 (0.398, 0.679)		Ref		Ref	
Male	0.667 (0.398, 0.795)		0.129	0.09, 0.17***	0.03	-0.03, 0.09
**Marital Status**		**0.0001**				
Never married	0.868 (0.868, 0.95)		Ref		Ref	
Married	0.646 (0.44. 0.718)		-0.22	-0.31, -0.13***	-0.11	-0.25, 0.03
Divorced/Separated	0.22 (0.031, 0.44)		-0.65	-0.79, -0.50***	-0.52	-0.71, -0.33***
Widowed	0.517 (0.268, 0.667)		-0.35	-0.46, -0.25***	-0.14	-0.30, -0.02
**Highest Education Level**		**0.0004**				
No formal education	0.531 (0.2745, 0.772)		Ref		Ref	
Primary	0.59 (0.299, 0.667)		0.28	0.04, 0.51*	-0.17	-0.34, -6.29*
JHS/JSS/Middle School	0.515 (0.237, 0.667)		0.2	-0.004, 0.40	-0.11	-0.26, 0.03
SHS/SSS/O-Level	0.625 (0.398, 0.667)		0.31	0.11, 0.51**	-0.13	-0.27, 0.01
Tertiary	0.667 (0.513, 0.868)		0.35	0.16, 0.55***	-0.05	-0.19, 0.08
**Religion**		**0.2321**				
Christianity	0.625 (0.398, 0.795)		Ref			
Islam	0.646 (0.398, 0.667)		0.42	-0.39, 0.58		
African Traditional	0.667 (0.667, 0.667)		0.16	-035, 0.65		
**Ethnicity**		**0.3154**				
Akan	0.59 (0.398, 0.679)		Ref			
Ewe	0.658 (0.398, 0.752)		0.07	-0.003, -0.14		
Ga/Dangme	0.667 (0.454, 0.795)		0.08	-0.60, 0.22		
Guan	0.59 (0.031, 0.795)		0	-0.14, 0.14		
**Diagnosed CNCD**		**0.0001**				
Cancer	-0.023 (-0.314, 0.361)		Ref		Ref	
Chronic Kidney Disease	0.667 (0.513, 0.752)		0.69	0.56, 0.82***	0.26	0.10, 0.42***
Diabetes	0.658 (0.398, 0.667)		0.68	0.55, 0.81***	0.28	0.11, 0.45***
Hypertension	0.6945 (0.594, 0.868)		0.69	0.58, 0.81***	0.35	0.19, 0.50***
Stroke	0.4855 (0.398, 0.59)		0.50	0.38, 0.62***	0.26	0.11, 0.40***
**Diagnosis Duration**		**0.0125**				
< 1 Year	0.517 (0.361, 0.667)		Ref		Ref	
1–5 Years	0.6415 (0.447, 0.7735)		0.11	0.04, 0.17***	0.005	-0.07, 0.08
6–10 Years	0.667 (0.0513, 0.752)		0.15	0.08, 022***	0.0003	-0.12, 0.12
10 + Years	0.646 (0.268, 0.862)		0.13	0.04, 0.21**	0.02	-0.11, 0.15
**Living With Comorbidities**		**0.0001**				
No	0.667 (0.44, 0.862)		Ref		Ref	
Yes	0.517 (0.299, 0.667)		-0.15	-0.20, -0.10***	-0.95	-0.15, -0.04***
**Comorbidities**		**0.0001**				
Arthritis	0.671 (0.671, 0.671)		Ref			
Bodily pains	0.139 (0.139, 0.139)		0.34	-0.32, 0.03		
Diabetes	0.454 (0.268, 0.646)		0.45	-0.06, 0.09		
Diabetes &Hypertension	0.166 (-0.143, 0.475)		0.61	0.65, 1.3		
Hypertension	0.517 (0.398, 0.667)		0.17	-0.08, 0.56		
Hernia	-0.143 (-0.143, -0.143)		0.34	0.56, 3.23		
Peptic ulcer	0.019 (0.019, 0.019)		0.3	0.40, 1.16		
Rheumatism	1 (1, 1)		0.10	-0.34, 0.41		
Stroke	0.5535 (0.513, 0.594)		0.56	-0.68, 0.56		
**Are you currently on any behavioural/lifestyle treatment?**		**0.0007**				
No	1 (1, 1)		Ref		Ref	
Yes	0.625 (0.398, 0.7505)		0	-0.16,0.16	0.12	0.03, 0.22**
**Recommended behavioural/lifestyle changes**						
**Dietary Changes**		**0.0001**				
No	0.44 (0.313, 0.517)		Ref		Ref	
Yes	0.667 (0.44, 0.795)		0.23	0.16, 0.29***	0.22	0.13, 0.31***
**Physical Activity**		**0.0058**				
No	0.679 (0.534, 0.918)		Ref			
Yes	0.602 (0.398, 0.735)		-0.08	-0.19, 0.03		
**Smoke Cessation**		**0.1788**				
No	0.625 (0.398, 0.749)		Ref			
Yes	0.765 (0.765, 0.765)		0.12	-0.26, 0.08		
**Alcohol Intake Moderation**		**0.2660**				
No	0.594 (0.398, 0.752)		Ref			
Yes	0.658 (0.534, 0.667)		0.06	-0.004, 0.13		

*p < 0.05 **p < 0.01 *** *p* < 0.001

## Discussion

We examined the health-related quality of life of adults living with CNCDs in the Ho Municipality. We found that the majority of our respondents reported having problems with all the dimensions of the EQ-5D-5L scale. The most affected dimension was the Anxiety/Depression (94%) dimension whereas the least affected was the Selfcare (59%) dimension. The results of the study also postulated that the marital status of respondents, their level of education, the specific CNCD they had been diagnosed with, their comorbidity status, and their dietary change behaviour predicted their level of HRQoL. The high levels of problems reported in this study are worrying as they may have negative implications on the general well-being of adults living with CNCDs as well as the achievement of the sustainable development goals (SDGs), specifically target 3.4 that aims to reduce premature mortality from non-communicable diseases by one-third by 2030.

In the current study, 63.7% of our respondents reported some problem in at least one of the dimensions measured. This proportion was similar to that found in another study which reported 64% of their respondents having problems in at least one of the dimensions [33]. In our study, the most affected dimension of HRQoL was the Anxiety/Depression dimension followed by the Pain/Discomfort dimension. 94% and 91% of our respondents reported some problems with these dimensions, respectively. This finding was consistent with the findings of other studies albeit with varying proportions [33–36]. The difference in proportions could likely be attributed to the distinct socio-cultural environments. However, it is worth noting that, of all these studies the Anxiety/Depression and Pain/Discomfort dimensions recur as the top two dimensions with the most problems reported. This underscores the need to incorporate mental health services and pain management into various CNCD treatments.

Our study found that the marital status of respondents has statistical significance with their HRQoL. We found that divorced/separated patients were less likely to report a high index of HRQoL when compared to patients who had never married. This finding was inversely reiterated by those of Han et al. [37] and Zhu et al. [38] which found that married individuals had a higher HRQoL. This was attributed to the fact that married or partnered individuals are likely to benefit from the companionship of their spouses or partners. This companionship is most likely to be lacking in the case of divorced/separated individuals.

Additionally, we found in our study that respondents’ HRQoL was predicted by their level of education. We found that respondents who reported having only primary-level education were less likely to report a high index for HRQoL than those with no formal education. This finding was inversely corroborated by the findings of other studies which postulated that the higher an individual’s level of education the higher their HRQoL is likely to be [39–42]. This could likely be attributed to the fact that individuals gain more health awareness with increasing levels of education which allows them to be more health conscious.

On the other hand, we observed that respondents’ specific CNCD diagnosis impacts their HRQoL. Respondents diagnosed of CKD, diabetes and hypertension were more likely to report a high HRQoL index as compared to those diagnosed with cancers. These findings were consistent with those by Tannor et al. [43] and Liang et al. [44] which similarly posit that the specific diagnosis of patients was impactful on their HRQoL. This may be attributed to the fact that many of these individuals are unable to reconcile with the reality of their diagnosis how they would invariably be on treatment for the rest of their life and how they may have to make drastic lifestyle adjustments to their new realities. All these point to the lack of health system support which could have seamlessly assisted these individuals with making these transitions. This, similarly, indicates the varying impact different CNCDs may have on an individual’s HRQoL which would require a disease-specific approach in management.

However, we found that respondents who reported living with comorbidities were less likely to report a high index for HRQoL. This finding was consistent with those of other studies which reported the negative impact of comorbidities on an individual’s HRQoL [45,46]. This underscores the need for a robust treatment regimen which accommodates other comorbidities of patients aside from their primary diagnosis to provide wholistic care which could improve patients’ HRQoL.

Furthermore, we discovered that respondents who were on behavioural/lifestyle treatment especially those who were recommended dietary change by their physician and nurses were more likely to report a high HRQoL index. This was in line with the findings of Qin et al. [47] which revealed in their study that respondents who consumed fast food, and sugar-sweetened beverages regularly were more likely to report a lower HRQoL. This highlights the importance of the role of dieticians and dietary education in the management of CNCDs.

## Strengths and limitations

In this study, we provided important insights into the impact of CNCDs on the quality of life of affected individuals, which was lacking in the status quo. By examining multiple dimensions of health, including physical, mental, and social well-being, the study identifies areas where interventions can be targeted to improve the quality of life of individuals with CNCDs. In addition, our use of quantile regression ensured that we robustly established the relationships that exist between our outcome and explanatory variables. However, a limitation of the study is that we relied on self-reported data, which may be subject to recall bias and social desirability bias. **Additionally, the use of a simple random sampling technique could potentially influence the interpretation of our results.** Despite these limitations, the study can provide valuable information for healthcare providers and policymakers to help improve the lives of those living with chronic non-communicable diseases.

## Conclusion

This study examined the HRQoL of persons living with CNCDs and found that more than half of the study respondents reported some problem in at least one of the HRQoL dimensions. With the “Anxiety/Depression” and “Pain/Discomfort” dimensions having the highest proportions of problems reported. Marital and comorbidity status as well as education were predictors of low HRQoL index. The above-stated phenomena could have negative effects on the treatment outcomes of persons living with CNCDs such as worsening of their conditions which could further perpetuate the high levels of morbidity and mortality due to CNCDs in Ghana. These have the potential to further undermine interventions put in place to reduce the incidence of CNCD mortality and morbidity as well as undercut Ghana’s attempt to achieve target 3.4 of the Sustainable Development Goals (SDGs) which seeks to reduce by one-third premature mortality from NCDs through prevention and treatment by 2030.

### Electronic supplementary material

Below is the link to the electronic supplementary material.

Supplementary Material 1

Supplementary Material 2

Supplementary Material 3

## Data Availability

All relevant data are within the manuscript and its Supporting Information files. Any further requests regarding the data used for this study could be made through the corresponding author.

## Abbreviations

aOR: Adjusted Odds Ratio
CKD: Chronic Kidney Disease
CNCDs: Chronic Non-Communicable Diseases
cOR: Crude Odds Ratio
HRQoL: Health-Related Quality of Life
HTH: Ho Teaching Hospital
JHS: Junior High School
JSS: Junior Secondary School
LMICs: Low- and Middle-Income Countries
QoL: Quality of Life
REC: Research Ethics Committee
SDGs: Sustainable Development Goals
SHS: Senior High School
SSA: Sub-Sahara Africa
SSS: Senior Secondary School
STROBE: Strengthening the Reporting of Observational Studies in Epidemiology
UHAS: University of Health and Allied Sciences

## References

[CR1] 1. United Nations. Transforming our world: The 2030 agenda for sustainable development. New York; 2015.

[CR2] 2. Hajat C, Stein E. The global burden of multiple chronic conditions: A narrative review. Prev Med Rep [Internet]. 2018 Dec 1 [cited 2022 Apr 14];12:284. Available from: /pmc/articles/PMC6214883/10.1016/j.pmedr.2018.10.008 PMC6214883 30406006

[CR3] 3. World Health Statistics. World Health Statistics 2020: monitoring health for the SDGs, sustainable development goals. Geneva; 2020.

[CR4] 4. Dugee O, Palam E, Dorjsuren B, Mahal A. Who is bearing the financial burden of non-communicable diseases in Mongolia? J Glob Health. 2018;8(1). 10.7189/jogh.08.010415 PMC5857203 29564086

[CR5] 5. Wang Q, Brenner S, Kalmus O, Banda HT, De Allegri M. The economic burden of chronic non-communicable diseases in rural Malawi: An observational study. BMC Health Serv Res. 2016 Sep 1;16(1). 10.1186/s12913-016-1716-8 PMC5007731 27582052

[CR6] 6. Ndubuisi NE. Noncommunicable Diseases Prevention In Low- and Middle-Income Countries: An Overview of Health in All Policies (HiAP). Inquiry [Internet]. 2021 [cited 2023 Mar 27];58. Available from: /pmc/articles/PMC8385577/10.1177/0046958020927885 PMC8385577 34420412

[CR7] 7. Gakidou E, Afshin A, Abajobir AA, Abate KH, Abbafati C, Abbas KM, et al. Global, regional, and national comparative risk assessment of 84 behavioural, environmental and occupational, and metabolic risks or clusters of risks, 1990–2016: a systematic analysis for the Global Burden of Disease Study 2016. Lancet [Internet]. 2017 Sep 16 [cited 2022 Apr 13];390(10100):1345–422. Available from: https://pubmed.ncbi.nlm.nih.gov/28919119/10.1016/S0140-6736(17)32366-8 PMC5614451 28919119

[CR8] 8. World Health Organization. Noncommunicable diseases country profiles 2018 [Internet]. Geneva; 2018 [cited 2021 Dec 15]. Available from: https://apps.who.int/iris/bitstream/handle/10665/274512/9789241514620-eng.pdf

[CR9] 9. Yuyun MF, Sliwa K, Kengne AP, Mocumbi AO, Bukhman G. Cardiovascular Diseases in Sub-Saharan Africa Compared to High-Income Countries: An Epidemiological Perspective. Glob Heart [Internet]. 2020 Feb 12 [cited 2023 Mar 27];15(1). Available from: /pmc/articles/PMC7218780/10.5334/gh.403 PMC7218780 32489788

[CR10] 10. WHO. Deaths from noncommunicable diseases on the rise in Africa [Internet]. 2022 [cited 2023 Mar 27]. Available from: https://www.afro.who.int/news/deaths-noncommunicable-diseases-rise-africa

[CR11] 11. Germain N, Aballéa S, Toumi M. Measuring health-related quality of life in young children: how far have we come? [Internet]. 2019 Jan 1 [cited 2022 Feb 5];7(1):1618661. 10.1080/20016689.2019.1618661 10.1080/20016689.2019.1618661 PMC6534256 31156762

[CR12] 12. Megari K. Quality of Life in Chronic Disease Patients. Health Psychol Res [Internet]. 2013 Sep 23 [cited 2022 Feb 3];1(3):27. Available from: /pmc/articles/PMC4768563/10.4081/hpr.2013.e27 PMC4768563 26973912

[CR13] 13. De Smedt D, Clays E, Annemans L, Pardaens S, Kotseva K, De Bacquer D. Self-reported health status in coronary heart disease patients: A comparison with the general population. European Journal of Cardiovascular Nursing [Internet]. 2015 Apr 1 [cited 2022 Feb 3];14(2):117–25. Available from: https://academic.oup.com/eurjcn/article/14/2/117/5932529 10.1177/1474515113519930 24434050

[CR14] 14. Xu RH, Cheung AWL, Wong ELY. Examining the health-related quality of life using EQ-5D-5L in patients with four kinds of chronic diseases from specialist outpatient clinics in Hong Kong SAR, China. Patient Prefer Adherence [Internet]. 2017 Sep 12 [cited 2022 Feb 3];11:1565. Available from: /pmc/articles/PMC5602472/10.2147/PPA.S143944 PMC5602472 28979104

[CR15] 15. Maniscalco L, Miceli S, Bono F, Matranga D. Self-Perceived Health, Objective Health, and Quality of Life among People Aged 50 and Over: Interrelationship among Health Indicators in Italy, Spain, and Greece. International Journal of Environmental Research and Public Health 2020, Vol 17, Page 2414 [Internet]. 2020 Apr 2 [cited 2022 Feb 2];17(7):2414. Available from: https://www.mdpi.com/1660-4601/17/7/2414/htm 10.3390/ijerph17072414 PMC7178192 32252321

[CR16] 16. Xiao M, Zhang F, Xiao N, Bu X, Tang X, Long Q. Health-Related Quality of Life of Hypertension Patients: A Population-Based Cross-Sectional Study in Chongqing, China. International Journal of Environmental Research and Public Health 2019, Vol 16, Page 2348 [Internet]. 2019 Jul 3 [cited 2022 Feb 2];16(13):2348. Available from: https://www.mdpi.com/1660-4601/16/13/2348/htm 10.3390/ijerph16132348 PMC6652141 31277210

[CR17] 17. Reba K, Argaw Z, Walle B, Gutema H. Health-related quality of life of patients with diagnosed type 2 diabetes in Felege Hiwot Referral Hospital, North West Ethiopia: A cross-sectional study. BMC Res Notes [Internet]. 2018 Aug 2 [cited 2022 Feb 2];11(1):1–6. Available from: https://link.springer.com/articles/10.1186/s13104-018-3625-x 10.1186/s13104-018-3625-x PMC6071393 30068392

[CR18] 18. Abegaz TM, Ayele AA, Gebresillassie BM. Health Related Quality of Life of Cancer Patients in Ethiopia. J Oncol. 2018;2018. 10.1155/2018/1467595 PMC5925207 29849628

[CR19] 19. Rosa EM, Tudge J. Urie Bronfenbrenner’s Theory of Human Development: Its Evolution From Ecology to Bioecology. J Fam Theory Rev. 2013 Dec;5(4):243–58.

[CR20] 20. Bronfenbrenner U. Making human beings human: bioecological perspectives on human development by Urie Bronfenbrenner. London: Sage; 2005. 1–372 p.

[CR21] 21. Konieczny M, Cipora E, Sygit K, Fal A. Quality of life of women with breast cancer and socio-demographic factors. Asian Pacific Journal of Cancer Prevention. 2020;21(1). 10.31557/APJCP.2020.21.1.185 PMC7294011 31983183

[CR22] 22. Arab-Zozani M, Hashemi F, Safari H, Yousefi M, Ameri H. Health-related quality of life and its associated factors in COVID-19 patients. Osong Public Health Res Perspect. 2020;11(5). 10.24171/j.phrp.2020.11.5.05 PMC7577388 33117634

[CR23] 23. Bélanger E, Ahmed T, Vafaei A, Curcio CL, Phillips SP, Zunzunegui MV. Sources of social support associated with health and quality of life: A cross-sectional study among Canadian and Latin American older adults. BMJ Open. 2016;6(6). 10.1136/bmjopen-2016-011503 PMC4932270 27354077

[CR24] 24. Von Elm E, Altman DG, Egger M, Pocock SJ, Gøtzsche PC, Vandenbrouckef JP. The Strengthening the Reporting of Observational Studies in Epidemiology (STROBE) Statement: Guidelines for reporting observational studies. Vol. 85, Bulletin of the World Health Organization. 2007. p. 867–72. 10.2471/BLT.07.045120 PMC2636253 18038077

[CR25] 25. Osei E, Amu H, Appiah-Kubi P, Konlan KD, Mumuni H, Orish VN, et al. Prevalence and predictors of selected risk factors of non-communicable diseases in Ghana: evidence from a sub-national survey. Journal of Global Health Science [Internet]. 2021 Nov 23 [cited 2022 Jan 8];3. Available from: 10.35500/jghs.2021.3.e13

[CR26] 26. Feng YS, Kohlmann T, Janssen MF, Buchholz I. Psychometric properties of the EQ-5D-5L: a systematic review of the literature. Quality of Life Research [Internet]. 2021 Mar 1 [cited 2023 Mar 8];30(3):647–73. Available from: https://link.springer.com/article/10.1007/s11136-020-02688-y 10.1007/s11136-020-02688-y PMC7952346 33284428

[CR27] 27. Jankowska A, Młyńczak K, Golicki D. Validity of EQ-5D-5L health-related quality of life questionnaire in self-reported diabetes: evidence from a general population survey. Health Qual Life Outcomes [Internet]. 2021 Dec 1 [cited 2023 Mar 8];19(1):1–11. Available from: https://hqlo.biomedcentral.com/articles/10.1186/s12955-021-01780-2 10.1186/s12955-021-01780-2 PMC8097836 33952271

[CR28] 28. Lartey ST, Si L, de Graaff B, Magnussen CG, Ahmad H, Campbell J, et al. Evaluation of the Association Between Health State Utilities and Obesity in Sub-Saharan Africa: Evidence From World Health Organization Study on Global AGEing and Adult Health Wave 2. Value in Health. 2019 Sep 1;22(9):1042–9. 10.1016/j.jval.2019.04.1925 31511181

[CR29] 29. Boutib A, Chergaoui S, Azizi A, Saad EM, Hilali A, Marfak IY, et al. Health-related quality of life during three trimesters of pregnancy in Morocco: cross-sectional pilot study. 2023 [cited 2023 Sep 25]; Available from: www.thelancet.com 10.1016/j.eclinm.2023.101837 PMC9932347 36816344

[CR30] 30. Shimels T, Kassu R, Bogale G, Bekele M, Getnet M. Health related quality of life of patients with chronic non-communicable diseases during the coronavirus pandemic in Ethiopia: a multi-facility study. 2020 [cited 2023 Mar 8]; Available from: https://www.researchsquare.com/article/rs-108488/latest.pdf

[CR31] 31. Yang F, Katumba KR, Roudijk B, Yang Z, Revill P, Griffin S, et al. Developing the EQ-5D-5L Value Set for Uganda Using the ‘Lite’ Protocol. Pharmacoeconomics [Internet]. 2022 Mar 1 [cited 2023 Nov 27];40(3):309–21. Available from: https://link.springer.com/article/10.1007/s40273-021-01101-x 10.1007/s40273-021-01101-x PMC8627844 34841471

[CR32] 32. EuroQol Research Foundation. EQ-5D-5L User Guide [Internet]. 2019 [cited 2023 Mar 27]. Available from: https://euroqol.org/publications/user-guides/

[CR33] 33. Barua L, Faruque M, Chowdhury HA, Banik PC, Ali L. Health-related quality of life and its predictors among the type 2 diabetes population of Bangladesh: A nation-wide cross-sectional study. J Diabetes Investig [Internet]. 2021 Feb 1 [cited 2023 Dec 31];12(2):277–85. Available from: https://onlinelibrary.wiley.com/doi/full/10.1111/jdi.13331 10.1111/jdi.13331 PMC7858106 32564501

[CR34] 34. Jackson IL, Isah A, Arikpo AO. Assessing health-related quality of life of people with diabetes in Nigeria using the EQ-5D-5L: a cross-sectional study. Sci Rep [Internet]. 2023 Dec 1 [cited 2023 Dec 31];13(1). Available from: /pmc/articles/PMC10728144/10.1038/s41598-023-49322-8 PMC10728144 38110447

[CR35] 35. Lin Y, Huang Y, Xi X. Association between lifestyle behaviors and health-related quality of life among primary health care physicians in China: A cross-sectional study. Front Public Health [Internet]. 2023 [cited 2023 Dec 31];11. Available from: /pmc/articles/PMC10030863/10.3389/fpubh.2023.1131031 PMC10030863 36969630

[CR36] 36. Namdeo MK, Verma S, Das Gupta R, Islam R, Nazneen S, Rawal LB. Depression and health-related quality of life of patients with type 2 diabetes attending tertiary level hospitals in Dhaka, Bangladesh. Glob Health Res Policy [Internet]. 2023 Dec 1 [cited 2023 Dec 31];8(1):1–14. Available from: https://ghrp.biomedcentral.com/articles/10.1186/s41256-023-00328-9 10.1186/s41256-023-00328-9 PMC10577997 37845742

[CR37] 37. Han KT, Park EC, Kim JH, Kim SJ, Park S. Is marital status associated with quality of life? Health Qual Life Outcomes [Internet]. 2014 Aug 8 [cited 2023 Dec 30];12(1):1–10. Available from: https://hqlo.biomedcentral.com/articles/10.1186/s12955-014-0109-0 10.1186/s12955-014-0109-0 PMC4148557 25104276

[CR38] 38. Zhu C, Tran PM, Leifheit EC, Spatz ES, Dreyer RP, Nyhan K, et al. Association of marital/partner status and patient-reported outcomes following myocardial infarction: a systematic review and meta-analysis. European Heart Journal Open [Internet]. 2023 Mar 1 [cited 2023 Dec 30];3(2):1–12. Available from: /pmc/articles/PMC10023828/10.1093/ehjopen/oead018 PMC10023828 36942107

[CR39] 39. Braveman P, Gottlieb L. The social determinants of health: it’s time to consider the causes of the causes. Public Health Rep [Internet]. 2014 [cited 2023 Dec 31];129 Suppl 2(Suppl 2):19–31. Available from: https://pubmed.ncbi.nlm.nih.gov/24385661/10.1177/00333549141291S206 PMC3863696 24385661

[CR40] 40. Siqeca F, Yip O, Mendieta MJ, Schwenkglenks M, Zeller A, De Geest S, et al. Factors associated with health-related quality of life among home-dwelling older adults aged 75 or older in Switzerland: a cross-sectional study. Health Qual Life Outcomes [Internet]. 2022 Dec 1 [cited 2023 Dec 31];20(1). Available from: /pmc/articles/PMC9773624/10.1186/s12955-022-02080-z PMC9773624 36544173

[CR41] 41. Gil-Lacruz M, Gil-Lacruz AI, Gracia-Pérez ML. Health-related quality of life in young people: The importance of education. Health Qual Life Outcomes [Internet]. 2020 Jun 16 [cited 2023 Dec 31];18(1):1–13. Available from: https://hqlo.biomedcentral.com/articles/10.1186/s12955-020-01446-5 10.1186/s12955-020-01446-5 PMC7298764 32546249

[CR42] 42. Alcañiz M, Solé-Auró A. Feeling good in old age: Factors explaining health-related quality of life. Health Qual Life Outcomes [Internet]. 2018 Mar 13 [cited 2023 Dec 31];16(1):1–9. Available from: https://hqlo.biomedcentral.com/articles/10.1186/s12955-018-0877-z 10.1186/s12955-018-0877-z PMC5851254 29534708

[CR43] 43. Tannor EK, Norman BR, Adusei KK, Sarfo FS, Davids MR, Bedu-Addo G. Quality of life among patients with moderate to advanced chronic kidney disease in Ghana - A single centre study. BMC Nephrol [Internet]. 2019 Apr 8 [cited 2023 Jan 3];20(1):1–10. Available from: https://bmcnephrol.biomedcentral.com/articles/10.1186/s12882-019-1316-z 10.1186/s12882-019-1316-z PMC6454740 30961570

[CR44] 44. Liang Z, Zhang T, Lin T, Liu L, Wang B, Fu AZ, et al. Health-related quality of life among rural men and women with hypertension: assessment by the EQ-5D-5L in Jiangsu, China. Quality of Life Research [Internet]. 2019 Aug 15 [cited 2023 Jan 3];28(8):2069–80. Available from: https://link.springer.com/article/10.1007/s11136-019-02139-3 10.1007/s11136-019-02139-3 30830645

[CR45] 45. Mannan A, Akter KM, Akter F, Chy NUHA, Alam N, Pinky SD, et al. Association between comorbidity and health-related quality of life in a hypertensive population: a hospital-based study in Bangladesh. BMC Public Health. 2022 Dec 1;22(1). 10.1186/s12889-022-12562-w PMC8793199 35081905

[CR46] 46. Saqlain M, Riaz A, Ahmed A, Kamran S, Bilal A, Ali H. Predictors of Health-Related Quality of Life Status Among Elderly Patients With Cardiovascular Diseases. Value Health Reg Issues. 2021 May 1;24:130–40. 10.1016/j.vhri.2020.11.003 33571727

[CR47] 47. Qin Z, Wang N, Ware RS, Sha Y, Xu F. Lifestyle-related behaviors and health-related quality of life among children and adolescents in China. Health Qual Life Outcomes. 2021 Dec 1;19(1). 10.1186/s12955-020-01657-w PMC7788787 33407589

