# Supplementary material for: Health-related quality of life among adults living with chronic non-communicable diseases in the Ho Municipality of Ghana: a health facility-based cross-sectional study
Source: BMC Public Health. 2024 Mar 6;24:725. doi: 10.1186/s12889-024-18143-3 (PMC10918919; doi:10.1186/s12889-024-18143-3)
Supplement: Supplementary file 1 — Supplementary Material 1 [file 12889_2024_18143_MOESM1_ESM.docx]

**Policy and practice**

**The Strengthening the Reporting of Observational Studies in Epidemiology (STROBE) Statement: guidelines for reporting observational studies***

Erik von Elm,a Douglas G Altman,b Matthias Egger,a,c Stuart J Pocock,d Peter C Gøtzsche e & Jan P Vandenbroucke f

for the STROBE Initiative

**Abstract** Much biomedical research is observational. The reporting of such research is often inadequate, which hampers the assessment of its strengths and weaknesses and of a study’s generalizability.The Strengthening the Reporting of Observational Studies in Epidemiology (STROBE) Initiative developed recommendations on what should be included in an accurate and complete report of an observational study. We defined the scope of the recommendations to cover three main study designs: cohort, case-control and cross-sectional studies. We convened a two-day workshop, in September 2004, with methodologists, researchers and journal editors to draft a checklist of items. This list was subsequently revised during several meetings of the coordinating group and in e-mail discussions with the larger group of STROBE contributors, taking into account empirical evidence and methodological considerations. The workshop and the subsequent iterative process of consultation and revision resulted in a checklist of 22 items (the STROBE Statement) that relate to the title, abstract, introduction, methods, results and discussion sections of articles. Eighteen items are common to all three study designs and four are specific for cohort, case-control, or cross-sectional studies. A detailed *Explanation and Elaboration* document is published separately and is freely available on the web sites of *PLoS Medicine, Annals of Internal Medicine and Epidemiology*. We hope that the STROBE Statement will contribute to improving the quality of reporting of observational studies.

Bulletin of the World Health Organization 2007;85:867–872.

***الرتجمة العربية لهذه الخالصة يف نهاية النص الكامل لهذه املقالة.*** *español. al traducción una facilita se artículo del final Al l’article. de fin la à figure résumé ce de français en traduction Une*

# Introduction

Many questions in medical research are investigated in observational studies.1 Much of the research into the cause of diseases relies on cohort, case-control or cross-sectional studies. Observational studies also have a role in research into the benefits and harms of medical interventions.2 Randomized trials cannot answer all important questions about a given intervention. For example, observational studies are more suitable to detect rare or late adverse effects of treatments, and are more likely to provide an indication of what is achieved in daily medical practice.3

Research should be reported transparently so that readers can follow what was

planned, what was done, what was found, and what conclusions were drawn. The credibility of research depends on a critical assessment by others of the strengths and weaknesses in study design, conduct and analysis. Transparent reporting is also needed to judge whether and how results can be included in systematic reviews.4,5 However, in published observational research important information is often missing or unclear. An analysis of epidemiological studies published in general medical and specialist journals found that the rationale behind the choice of potential confounding variables was often not reported.6 Only few reports of case-control studies in psychiatry explained the methods used to identify cases and controls.7 In a survey

of longitudinal studies in stroke research, 17 of 49 articles (35%) did not specify the eligibility criteria.8 Others have argued that without sufficient clarity of reporting, the benefits of research might be achieved more slowly,9 and that there is a need for guidance in reporting observational studies.10,11

Recommendations on the report- ing of research can improve reporting quality. The Consolidated Standards of Reporting Trials (CONSORT) Statement was developed in 1996 and revised five years later.12 Many medical journals supported this initiative,13 which has helped to improve the quality of reports of randomized trials.14,15 Similar initiatives have followed for other research areas –

e.g. for the reporting of meta-analyses

* In order to encourage dissemination of the STROBE Statement, this paper will also be published and made freely available by *Annals of Internal Medicine, BMJ, Epidemiology, The Lancet, and PLoS Medicine and Preventive Medicine*. The authors jointly hold the copyright of this paper. For details on further use, see STROBE web site (http://www.strobe-statement.org).

a Institute of Social and Preventive Medicine (ISPM), University of Bern, Finkenhubelweg 11, CH-3012 Bern, Switzerland. Correspondence to Erik von Elm (e-mail: strobe@ispm.unibe.ch).

b Centre for Statistics in Medicine, University of Oxford, Oxford, England.

c Department of Social Medicine, University of Bristol, Bristol, England.

d London School of Hygiene and Tropical Medicine, University of London, London, England.

e Nordic Cochrane Centre, Copenhagen, Denmark.

f Department of Clinical Epidemiology, Leiden University Hospital, Leiden, the Netherlands. doi: 10.2471/BLT.**07.045120**

(*Submitted: 20 June 2007 – Final revised version submitted: 13 August 2007 – Accepted: 20 August 2007* )

of randomized trials16 or diagnostic studies.17 We established a network of methodologists, researchers and journal editors to develop recommendations for the reporting of observational research: the Strengthening the Reporting of Observational Studies in Epidemiology (STROBE) Statement.

# Aims and use of the STROBE Statement

The STROBE Statement is a checklist of items that should be addressed in articles reporting on the three main study designs of analytical epidemiology: cohort, case- control and cross-sectional studies. The intention is solely to provide guidance on how to report observational research well: these recommendations are not prescriptions for designing or conducting studies. Also, while clarity of reporting is a prerequisite to evaluation, the checklist is not an instrument to evaluate the quality of observational research.

Here we present the STROBE State-

ment and explain how it was developed. In a detailed companion paper, the *Explanation and Elaboration* article,18–20 we justify the inclusion of the different checklist items, and give methodological background and published examples of what we consider transparent report- ing. We strongly recommend using the STROBE checklist in conjunction with the explanatory article, which is available freely on the web sites of *PLoS Medicine* (www.plosmedicine.org), Annals of Internal Medicine (www.annals.org) and *Epidemiology* (www.epidem.com).

# Development of the STROBE Statement

We established the STROBE Initiative in 2004, obtained funding for a work- shop and set up a web site (www.strobe- statement.org). We searched textbooks, bibliographic databases, reference lists and personal files for relevant material, including previous recommendations, empirical studies of reporting and arti- cles describing relevant methodological research. Because observational research makes use of many different study de- signs, we felt that the scope of STROBE had to be clearly defined early on. We decided to focus on the three study designs that are used most widely in ana- lytical observational research: cohort, case-control and cross-sectional studies.

We organized a two-day workshop in Bristol, the United Kingdom, in Sep- tember 2004. Twenty-three individu- als attended this meeting, including editorial staff from *Annals of Internal Medicine*, *BMJ*, *Bulletin of the World Health Organization*, *International Jour- nal of Epidemiology*, *JAMA*, *Preventive Medicine* and *The Lancet* as well as epi- demiologists, methodologists, statisti- cians and practitioners from Europe and North America. Written contributions were sought from 10 other individuals who declared an interest in contribut- ing to STROBE, but could not attend. Three working groups identified items deemed to be important to include in checklists for each type of study. A pro- visional list of items prepared in advance (available from our web site) was used to facilitate discussions. The three draft checklists were then discussed by all participants and, where possible, items were revised to make them applicable to all three study designs. In a final plenary session, the group decided on the strat- egy for finalizing and disseminating the STROBE Statement.

After the workshop we drafted a combined checklist including all three designs and made it available on our web site. We invited participants and additional scientists and editors to comment on this draft checklist. We subsequently published three revisions on the web site, and two summaries of comments received and changes made. During this process the coordinating group (i.e. the authors of the present paper) met on eight occasions for one or two days, and held several telephone conferences to revise the checklist and to prepare the present paper and the *Explanation and Elaboration* paper.18–20 The coordinating group invited three additional co-authors with method- ological and editorial expertise to help write the *Explanation and Elaboration* paper, and sought feedback from more than 30 people, who are listed at the end of this paper. We allowed several weeks for comments on subsequent drafts of the paper and reminded collaborators about deadlines by e-mail.

# STROBE components

The STROBE Statement is a checklist of 22 items that we consider essential for good reporting of observational studies (see Table 1). These items relate to the article’s title and abstract (item 1), the

introduction (items 2 and 3), methods

(items 4–12), results (items 13–17), discussion sections (items 18–21) and other information (item 22 on fund- ing). Eighteen items are common to all three designs, while four (items 6, 12, 14 and 15) are design-specific, with dif- ferent versions for all or part of the item. For some items (indicated by footnote a), information should be given separately for cases and controls in case-control studies, or exposed and unexposed groups in cohort and cross-sectional studies. Although presented here as a single checklist, separate checklists are available for each of the three study de- signs on the STROBE web site.

# Implications and limitations

The STROBE Statement was developed to assist authors when writing up ana- lytical observational studies, to support editors and reviewers when considering such articles for publication, and to help readers when critically appraising published articles. We developed the checklist through an open process, tak- ing into account the experience gained with previous initiatives, in particular CONSORT. We reviewed the relevant empirical evidence as well as method- ological work, and subjected consecutive drafts to an extensive iterative process of consultation. The checklist presented here is thus based on input from a large number of individuals with diverse backgrounds and perspectives. The comprehensive explanatory article,18–20 which is intended for use alongside the checklist, also benefited greatly from this consultation process.

Observational studies serve a wide range of purposes, on a continuum from the discovery of new findings to the confirmation or refutation of previous findings.18–20 Some studies are essentially exploratory and raise interesting hypotheses. Others pursue clearly de- fined hypotheses in available data. In yet another type of studies, the collection of new data is planned carefully on the basis of an existing hypothesis. We believe the present checklist can be useful for all these studies, since the readers always need to know what was planned (and what was not), what was done, what was found, and what the results mean. We acknowledge that STROBE is currently limited to three main observational study designs. We would welcome extensions that adapt

| Table 1. **The STROBE Statement: a checklist of items that should be addressed in reports of observational studies** | | |
| --- | --- | --- |
| **Item** | **Item number** | **Recommendation** |
| **Title and abstract** | 1 | (a) Indicate the study’s design with a commonly used term in the title or the abstract |
|  |  | (b) Provide in the abstract an informative and balanced summary of what was done and what was found |
| **Introduction** |  |  |
| **Background/rationale** | 2 | Explain the scientific background and rationale for the investigation being reported |
| **Objectives** | 3 | State specific objectives, including any pre-specified hypotheses |
| **Methods** |  |  |
| **Study design** | 4 | Present key elements of study design early in the paper |
| **Setting** | 5 | Describe the setting, locations and relevant dates, including periods of recruitment, exposure, follow-up and data collection |
| **Participants** | 6 | (a) *Cohort study* – Give the eligibility criteria, and the sources and methods of selection of participants. Describe methods of follow-up  *Case-control study* – Give the eligibility criteria, and the sources and methods of case ascertainment and control selection. Give the rationale for the choice of cases and controls *Cross-sectional study* – Give the eligibility criteria, and the sources and methods of selection of participants |
|  |  | (b) *Cohort study* – For matched studies, give matching criteria and number of exposed and unexposed  *Case-control study* – For matched studies, give matching criteria and the number of controls per case |
| **Variables** | 7 | Clearly define all outcomes, exposures, predictors, potential confounders and effect modifiers. Give diagnostic criteria, if applicable |
| **Data sources/measurement** | 8a | For each variable of interest, give sources of data and details of methods of assessment (measurement). Describe comparability of assessment methods if there is more than one group |
| **Bias** | 9 | Describe any efforts to address potential sources of bias |
| **Study size** | 10 | Explain how the study size was arrived at |
| **Quantitative variables** | 11 | Explain how quantitative variables were handled in the analyses. If applicable, describe which groupings were chosen and why |
| **Statistical methods** | 12 | (a) Describe all statistical methods, including those used to control for confounding |
|  |  | (b) Describe any methods used to examine subgroups and interactions |
|  |  | (c) Explain how missing data were addressed |
|  |  | (d) *Cohort study* – If applicable, explain how loss to follow-up was addressed  *Case-control study* – If applicable, explain how matching of cases and controls was addressed *Cross-sectional study* – If applicable, describe analytical methods taking account of sampling strategy |
|  |  | (e) Describe any sensitivity analyses |
| **Results** |  |  |
| **Participants** | 13^a^ | (a) Report the numbers of individuals at each stage of the study – e.g. numbers potentially eligible, examined for eligibility, confirmed eligible, included in the study, completing follow- up and analyzed |
|  |  | (b) Give reasons for non-participation at each stage |
|  |  | (c) Consider use of a flow diagram |
| **Descriptive data** | 14^a^ | (a) Give characteristics of study participants (e.g. demographic, clinical, social) and information on exposures and potential confounders |
|  |  | (b) Indicate the number of participants with missing data for each variable of interest |
|  |  | (c) *Cohort study* – Summarize follow-up time (e.g. average and total amount) |
| **Outcome data** | 15^a^ | *Cohort study* – Report numbers of outcome events or summary measures over time  *Case-control study* – Report numbers in each exposure category, or summary measures of exposure  *Cross-sectional study* – Report numbers of outcome events or summary measures |

(Table 1, cont.)

| **Item** | **Item number** | **Recommendation** |
| --- | --- | --- |
| **Main results** | 16 | (a) Give unadjusted estimates and, if applicable, confounder-adjusted estimates and their precision (e.g. 95% confidence interval). Make clear which confounders were adjusted for and why they were included |
|  |  | (b) Report category boundaries when continuous variables were categorized |
|  |  | (c) If relevant, consider translating estimates of relative risk into absolute risk for a meaningful time period |
| **Other analyses** | 17 | Report other analyses done – e.g. analyses of subgroups and interactions, and sensitivity analyses |
| **Discussion** |  |  |
| **Key results** | 18 | Summarize key results with reference to study objectives |
| **Limitations** | 19 | Discuss limitations of the study, taking into account sources of potential bias or imprecision. Discuss both direction and magnitude of any potential bias |
| **Interpretation** | 20 | Give a cautious overall interpretation of results considering objectives, limitations, multiplicity of analyses, results from similar studies, and other relevant evidence |
| **Generalizability** | 21 | Discuss the generalizability (external validity) of the study results |
| **Other information** |  |  |
| **Funding** | 22 | Give the source of funding and the role of the funders for the present study and, if applicable, for the original study on which the present article is based |
| a Give such information separately for cases and controls in case-control studies, and, if applicable, for exposed and unexposed groups in cohort and cross-sectional studies.  An *Explanation and Elaboration* article18–20 discusses each checklist item, and gives methodological background and published examples of transparent reporting. The STROBE checklist is best used in conjunction with this article (freely available on the web sites of *PLoS Medicine*, *Annals of Internal Medicine* and *Epidemiology* ). Separate versions of the checklist for cohort, case-control and cross-sectional studies are available on the STROBE web site. | | |

the checklist to other designs – e.g. case-crossover studies or ecological studies – and also to specific topic areas. Four extensions are now available for the CONSORT Statement.21–24 A first extension to STROBE is underway for gene–disease association studies: the STROBE Extension to Genetic Association studies (STREGA) initiative.25 We ask those who aim to develop extensions of the STROBE Statement to contact the coordinating group first to avoid duplication of effort.

The STROBE Statement should not be interpreted as an attempt to prescribe the reporting of observational research in a rigid format. The checklist items should be addressed in sufficient detail and with clarity somewhere in an article, but the order and format for presenting information depends on author preferences, journal style and the traditions of the research field. For instance, we discuss the reporting of results under several separate items, while recognizing that authors might address several items within a single section of text or in a table. Also, item 22, on the source of funding and the role of funders, could be addressed in an appendix or in the methods section of the article. We do not aim at standardizing reporting. Authors of randomized clinical

trials were asked by an editor of a specialist medical journal to “CONSORT” their manuscripts on submission.26 We believe that manuscripts should not be “STROBEd”, in the sense of regulating style or terminology. We encourage authors to use narrative elements, including the description of illustrative cases, to complement the essential information about their study, and to make their articles an interesting read.27

We emphasize that the STROBE Statement was not developed as a tool for assessing the quality of published observational research. Such instruments have been developed by other groups and were the subject of a recent systematic review.28 In the *Explanation and Elaboration* paper, we used several examples of good reporting from studies whose results were not confirmed in further research – the important feature was the good reporting, not whether the research was of good quality. However, if STROBE is adopted by authors and journals, issues such as confounding, bias and generalizability could become more transparent, which might help temper the over-enthusiastic reporting of new findings in the scientific community and popular media,29 and improve the methodology of studies in the long

term. Better reporting may also help to have more informed decisions about when new studies are needed and what they should address.

We did not undertake a compre- hensive systematic review for each of the checklist items and sub-items, or do our own research to fill gaps in the evidence base. Further, although no one was excluded from the process, the composition of the group of contributors was influenced by existing networks and was not representative in terms of geography (it was dominated by contributors from Europe and North America) and probably was not representative in terms of research interests and disciplines. We stress that STROBE and other recommendations on the reporting of research should be seen as evolving documents that require continual assessment, refinement, and, if necessary, change. We welcome suggestions for the further dissemination of STROBE – e.g. by re-publication of the present article in specialist journals and in journals published in other languages. Groups or individuals who intend to translate the checklist to other languages should consult the coordinating group beforehand. We will revise the checklist in the future, taking into account comments, criticism, new evidence and experience

from its use. We invite readers to submit their comments via the STROBE web site (www.strobe-statement.org). ■

#### Acknowledgments

The following individuals have contributed to the content and elaboration of the STROBE Statement: Douglas G Altman, Maria Blettner, Paolo Boffetta, Hermann Brenner, Geneviève Chêne, Cyrus Cooper, George Davey-Smith, Erik von Elm, Matthias Egger, France Gagnon, Peter C Gøtzsche, Philip Greenland, Sander Greenland, Claire Infante-Rivard, John Ioannidis, Astrid James, Giselle Jones, Bruno Ledergerber, Julian Little, Margaret May, David Moher, Hooman Momen, Alfredo

Morabia, Hal Morgenstern, Cynthia D Mulrow, Fred Paccaud, Stuart J Pocock, Charles Poole, Drummond Rennie, Martin Röösli, Dietrich Rothenbacher, Kenneth Rothman, Caroline Sabin, Willi Sauerbrei, Lale Say, James J Schlesselman, Jonathan Sterne, Holly Syddall, Jan P Vandenbroucke, Ian White, Susan Wieland, Hywel Williams, Guang Yong Zou.

The workshop was funded by the European Science Foundation (ESF). Additional funding was received from the Medical Research Council Health Services Collaboration and the National Health Services Research and Development Meth- odology Programme. We are grateful to Gerd Antes, Kay Dickersin, Shah Ebrahim

and Richard Lilford for supporting the STROBE Initiative. We are grateful to the following institutions that have hosted working meetings of the coordinating group: Institute of Social and Preventive Medicine (ISPM), University of Bern, Bern, Switzerland; Department of Social Medicine, University of Bristol, Bristol, the United Kingdom; London School of Hygiene and Tropical Medicine, London, the United Kingdom; Nordic Cochrane Centre, Copenhagen, Denmark; and Centre for Statistics in Medicine, Oxford, the United Kingdom. We are grateful to six reviewers who provided helpful comments on a previous draft of this paper.

**Competing interests:** None declared.

## Résumé

### Déclaration de l’Initiative STROBE (Strengthening the Reporting of Observational Studies in Epidemiology) : recommandations pour l’élaboration des rapports d’études observationnelles

Les recherches menées dans le domaine biomédical sont pour une grande part de nature observationnelle. Les rapports de ces études sont souvent inadéquats, ce qui empêche l’évaluation des points forts et des défauts de l’étude et sa généralisation. L’Initiative STROBE (Strengthening the Reporting of Observational Studies in Epidemiology) a élaboré des recommandations sur ce que doit être le contenu d’un rapport complet et précis d’étude observationnelle. Nous avons défini le champ d’application de ces recommandations comme couvrant les trois principaux types d’étude, à savoir les études de cohorte, cas-témoins et transversales. En septembre 2004, dans le cadre d’un atelier de deux jours, nous avons invité des méthodologistes, des chercheurs et des éditeurs de revue à élaborer une liste de points à considérer. Cette liste a ensuite été révisée dans le cadre de plusieurs réunions du groupe coordinateur et de discussions par

courrier électronique avec le groupe plus large des contributeurs à l’Initiative STROBE, en tenant compte de résultats empiriques et de considérations méthodologiques. L’atelier et le processus itératif ultérieur de consultation et de révision ont débouché sur une liste de contrôle en 22 points (la déclaration STROBE) concernant le titre, le résumé, l’introduction, la méthodologie et les parties Résultats et Discussion des articles. Dix-huit points s’appliquent collectivement aux trois types d’étude et quatre spécifiquement aux études de cohortes, cas-témoins ou transversales. Un document intitulé *Explanation and Elaboration* est publié séparément et accessible gratuitement sur les sites Internet *PloS Medicine, Annals of Internal Medicine* et *Epidemiology*. Nous espérons que la déclaration STROBE contribuera à l’amélioration de la qualité des rapports d’étude observationnelle.

## Resumen

### Declaración de la Iniciativa STOBE (Strengthening the Reporting of Observational Studies in Epidemiology): directrices para informar sobre los estudios observacionales

Gran parte de la investigación biomédica es de tipo observacional, pero la información difundida sobre esas investigaciones es a menudo insuficiente, lo que dificulta la evaluación de sus puntos fuertes y débiles y de su generalizabilidad de las conclusiones. En el marco de la iniciativa de Fortalecimiento de la Notificación de los Estudios Observacionales en Epidemiología (STROBE), se formularon recomendaciones sobre lo que debería contener una notificación precisa de un estudio observacional. Decidimos limitar el alcance de las recomendaciones a tres grandes modalidades de estudio: estudios de cohortes, estudios de casos y controles, y estudios transversales. En septiembre de 2004 organizamos un taller de dos días con metodólogos, investigadores y editores de revistas para elaborar una lista de verificación de distintos puntos. Esta lista fue revisada posteriormente en varias reuniones del grupo de coordinación y en discusiones mantenidas por e-mail

con los principales participantes en STROBE, teniendo en cuenta la evidencia empírica y diversas consideraciones metodológicas. El taller y el posterior proceso iterativo de consulta y revisión desembocaron en una lista de verificación de 22 puntos (la declaración STROBE) que guardan relación con el título, el resumen, la introducción y las secciones de métodos, resultados y discusión de los artículos. Dieciocho puntos son comunes a las tres modalidades de estudio, y cuatro se refieren específicamente a los estudios de cohortes, de casos y controles o transversales. Se ha publicado separadamente un documento de *Explicación y elaboración* al que puede accederse libremente en los sitios web de *PLoS Medicine, Annals of Internal Medicine* y *Epidemiology*. Esperamos que la declaración STROBE contribuya a mejorar la calidad de la notificación de los estudios observacionales.

**ملخص**

**تعزيز عملية إعداد تقارير الدراسات القامئة عىل املراقبة يف مجال الوبائيات: بيان سرتوب :STROBE دالئل إرشادية حول إعداد تقارير**

**الدراسات القامئة عىل املراقبة**

اإللكرتونية بني املجموعة األكرب من املشاركني يف بيان سرتوب STROBE، مع أخذ البينات التجريبية واالعتبارات املنهاجية يف الحسبان. وقد متخضت الحلقة العملية وما تالها من عمليات تشاور وتنقيح متكررة، عن قامئة تفقدية تضم 22 بنداً (بيان سرتوب )STROBE وهي ذات عالقة بعنوان

وخالصة ومقدمة وطرق ونتائج البحث وأقسام املناقشة الخاصة مبقاالته. وتشتمل القامئة عىل مثانية عرش بنداً مشرتكاً بني جميع أشكال الدراسات البحثية الثالثة، بينام تعد البنود األربعة الباقية بنوداً نوعية خاصة بالدراسات األترابية، أو الحاالت والشواهد، أو العرضية. وهناك وثيقة منفصلة تشمل رشحاً وتفصيال موسعني يف هذا املجال، منشورة عىل شبكة اإلنرتنت، وهي متاحة مجاناً عىل موقعي Medicine PLoS Medicine Internal of Annals و.Epidemiology ويأمل الباحثون يف أنُيسهم بيان سرتوب STROBE يف

تحسني نوعية التقارير املعدة عن الدراسات القامئة عىل املراقبة.

يقوم العديد من األبحاث الطبية البيولوجية عىل املراقبة، وغالباً ما يصعب إعداد التقارير عن تلك الدراسات، مام يعوق تقيـيم مواضع القوة ومواطن الضعف يف هذه األبحاث، وكذلك إمكانية التعميم فيها. ولقد نشأت عن مبادرة تعزيز عملية إعداد تقارير الدراسات القامئة عىل املراقبة يف مجال الوبائيات توصيات حول ما ينبغي أن يضمه تقرير دقيق وكامل عن إحدى الدراسات القامئة عىل املراقبة. وقدم الباحثون تعريفاً لنطاق هذه التوصيات

يغطي ثالثة أشكال رئيسية من الدراسات البحثية هي: الدراسات األترابية، ودراسات الحاالت والشواهد، والدراسات العرضية. وعقد الباحثون حلقة

عملية مدتها يومان يف أيلول/سبتمرب 2004، حرضها منهاجيون، وباحثون، ومحررو مجالت طبية، لوضع مسودة لقامئة تفقدية للبنود التي ينبغي أن تتضمنها مثل تلك التقارير. وخضعت هذه القامئة الحقاً للتنقيح يف عدة اجتامعات عقدتها املجموعة التنسيقية، وكذلك من خالل تبادل الرسائل

## References

1. Glasziou P, Vandenbroucke JP, Chalmers I. Assessing the quality of research.

*BMJ* 2004;328:39-41.

1. Black N. Why we need observational studies to evaluate the effectiveness of health care. *BMJ* 1996;312:1215-8.
2. Papanikolaou PN, Christidi GD, Ioannidis JP. Comparison of evidence on harms of medical interventions in randomized and nonrandomized studies. *CMAJ* 2006;174:635-41.
3. Jüni P, Altman DG, Egger M. Systematic reviews in health care: Assessing the quality of controlled clinical trials. *BMJ* 2001;323:42-6.
4. Egger M, Schneider M, Davey Smith G. Spurious precision? Meta-analysis of observational studies. *BMJ* 1998;316:140-4.
5. Pocock SJ, Collier TJ, Dandreo KJ, de Stavola BL, Goldman MB, Kalish LA, et al. Issues in the reporting of epidemiological studies: a survey of recent practice. *BMJ* 2004;329:883.
6. Lee W, Bindman J, Ford T, Glozier N, Moran P, Stewart R, et al. Bias in psychiatric case-control studies: literature survey. *Br J Psychiatry* 2007; 190:204-9.
7. Tooth L, Ware R, Bain C, Purdie DM, Dobson A. Quality of reporting of observational longitudinal research. *Am J Epidemiol* 2005;161:280-8.
8. Bogardus ST Jr, Concato J, Feinstein AR. Clinical epidemiological quality in molecular genetic research: the need for methodological standards. *JAMA* 1999;281:1919-26.
9. Guidelines for documentation of epidemiologic studies. Epidemiology Work Group of the Interagency Regulatory Liaison Group. *Am J Epidemiol* 1981;114:609-13.
10. Rennie D. CONSORT revised – improving the reporting of randomized trials.

*JAMA* 2001;285:2006-7.

1. Moher D, Schulz KF, Altman DG. The CONSORT statement: revised recommendations for improving the quality of reports of parallel-group randomised trials. *Lancet* 2001;357:1191-4.
2. Moher D, Altman DG, Schulz KF, Elbourne DR. Opportunities and challenges for improving the quality of reporting clinical research: CONSORT and beyond. *CMAJ* 2004;171:349-50.
3. Plint AC, Moher D, Morrison A, Schulz K, Altman DG, Hill C, et al. Does the CONSORT checklist improve the quality of reports of randomised controlled trials? A systematic review. *Med J Aust* 2006;185:263-7.
4. Egger M, Jüni P, Bartlett C. Value of flow diagrams in reports of randomized controlled trials. *JAMA* 2001;285:1996-9.
5. Moher D, Cook DJ, Eastwood S, Olkin I, Rennie D, Stroup DF. Improving the quality of reports of meta-analyses of randomised controlled trials: the QUOROM statement. Quality of reporting of meta-analyses. *Lancet* 1999; 354:1896-900.
6. Bossuyt PM, Reitsma JB, Bruns DE, Gatsonis CA, Glasziou PP, Irwig LM, et al. Towards complete and accurate reporting of studies of diagnostic accuracy: The STARD Initiative. *Ann Intern Med* 2003;138:40-4.
7. Vandenbroucke JP, von Elm E, Altman DG, Gøtzsche PC, Mulrow CD, Pocock SJ, et al. Egger M for the STROBE initiative. Strengthening the Reporting

of Observational Studies in Epidemiology (STROBE): Explanation and Elaboration. *PLoS Med* 2007;4: e297. doi:10.1371/journal.pmed.0040297

1. Vandenbroucke JP, von Elm E, Altman DG, Gøtzsche PC, Mulrow CD, Pocock SJ, Poole C, Schlesselman JJ, Egger M for the STROBE initiative. Strengthening the Reporting of Observational Studies in Epidemiology (STROBE): Explanation and Elaboration. *Annals of Intern Med* 2007. In press.
2. Vandenbroucke JP von Elm E. Altman DG, Gøtzsche PC, Mulrow CD, Pocock SJ, Poole C, Schlesselman JJ, Egger M for the STROBE initiative. Strengthening the Reporting of Observational Studies in Epidemiology (STROBE): Explanation and Elaboration. *Epidemiology* 2007. In press.
3. Ioannidis JP, Evans SJ, Gøtzsche PC, O’Neill RT, Altman DG, Schulz K, et al. Better reporting of harms in randomized trials: an extension of the CONSORT statement. *Ann Intern Med* 2004;141:781-8.
4. Campbell MK, Elbourne DR, Altman DG. CONSORT statement: extension to cluster randomised trials. *BMJ* 2004;328:702-8.
5. Piaggio G, Elbourne DR, Altman DG, Pocock SJ, Evans SJ. Reporting of noninferiority and equivalence randomized trials: an extension of the CONSORT statement. *JAMA* 2006;295:1152-60.
6. Gagnier JJ, Boon H, Rochon P, Moher D, Barnes J, Bombardier C. Reporting randomized, controlled trials of herbal interventions: an elaborated CONSORT statement. *Ann Intern Med* 2006;144:364-7.
7. Ioannidis JP, Gwinn M, Little J, Higgins JP, Bernstein JL, Boffetta P, et al. A road map for efficient and reliable human genome epidemiology. *Nat Genet* 2006;38:3-5.
8. Ormerod AD. CONSORT your submissions: an update for authors. *Br J Dermatol* 2001;145:378-9.
9. Schriger DL. Suggestions for improving the reporting of clinical research: the role of narrative. *Ann Emerg Med* 2005;45:437-43.
10. Sanderson S, Tatt ID, Higgins JP. Tools for assessing quality and susceptibility to bias in observational studies in epidemiology: a systematic review and annotated bibliography. *Int J Epidemiol*. 2007;36:666-76.
11. Bartlett C, Sterne J, Egger M. What is newsworthy? Longitudinal study

of the reporting of medical research in two British newspapers. *BMJ* 2002; 325:81-4.
