# Supplementary material for: Health-related quality of life among adults living with chronic non-communicable diseases in the Ho Municipality of Ghana: a health facility-based cross-sectional study
Source: BMC Public Health. 2024 Mar 6;24:725. doi: 10.1186/s12889-024-18143-3 (PMC10918919; doi:10.1186/s12889-024-18143-3)
Supplement: Supplementary file 2 — Supplementary Material 2 [file 12889_2024_18143_MOESM2_ESM.docx]

## Additional File 2 – Data Collection Instruments

**DATA COLLECTION INSTRUMENT I (QUESTIONNAIRE)**

**UNIVERSITY OF HEALTH AND ALLIED SCIENCES, HO**

**F. N. BINKA SCHOOL OF PUBLIC HEALTH**

This questionnaire is designed to assess the health-related quality of life among chronic non-communicable disease patients in the Ho Municipality. All information given on this questionnaire will be held confidential and used only for the purpose of the study.

| **For Official use only** | | | |
| --- | --- | --- | --- |
| QUESTIONNAIRE NO.: _______________ | | DATE OF INTERVIEW: ___/___/______ | |
|  |  |  |  |
| NAME OF INTERVIEWER: _______________________________ | | |  |

**Section 1: SOCIO-DEMOGRAPHIC CHARACTERISTICS**

| Code | Variables | Responses |  |
| --- | --- | --- | --- |
| Age | What is your age? (In completed years) | _______ |  |
| Sex | Sex of respondent? | 1. Male 2. Female | [ ]  [ ] |
| MarStat | What is your marital status? | 1. Never Married 2. Married 3. Divorced/Separated 4. Widowed | [ ]  [ ]  [ ]  [ ] |
| Edu_Level | What is your highest Educational Level? | 1. No Formal Education 2. Primary 3. JHS/JSS/Middle 4. SHS/SSS/O-Level 5. Tertiary | [ ]  [ ]  [ ]  [ ]  [ ] |
| Religion | What is your religion? | 1. Christianity 2. Islam 3. African Traditional 4. Other (specify) __________ | [ ]  [ ]  [ ]  [ ] |
| Ethnicity | What is your ethnicity? | 1. Akan 2. Ewe 3. Guan 4. Ga/Dangme 5. Mole-Dagbani 6. Other (specify) | [ ]  [ ]  [ ]  [ ]  [ ]  [ ] |
| Diagnosed CNCD | What chronic noncommunicable disease have you been diagnosed with? | ___________________ |  |
| Duration | How long have you been diagnosed with this condition? | ___________________ |  |
| Comorbidity | Are you currently living with any comorbidity? | 1. No 2. Yes   If yes please specify ­­­­­­­­______________ | [ ]  [ ] |

**Section 2: Health-related Quality of Life**

This Section of the questionnaire adopts the EQ-5D-5L instrument from the EuroQoL Group to measure the health-related quality of life.

| Under each heading, please mark the ONE box that best describes your health TODAY. |
| --- |

| **HQ001** | **MOBILITY (WALKING)** |  |
| --- | --- | --- |
|  | I have no problems in walking | **[ ]** |
|  | I have slight problems in walking | **[ ]** |
|  | I have moderate problems in walking | **[ ]** |
|  | I have severe problems in walking | **[ ]** |
|  | I am unable to walk | **[ ]** |
|  | I have no problems in walking | **[ ]** |
|  |  |  |
| **HQ002** | **SELF-CARE** |  |
|  | I have no problems washing or dressing myself | **[ ]** |
|  | I have slight problems washing or dressing myself | **[ ]** |
|  | I have moderate problems washing or dressing myself | **[ ]** |
|  | I have severe problems washing or dressing myself | **[ ]** |
|  | I am unable to wash or dress myself | **[ ]** |
|  |  |  |
| **HQ003** | **REGULAR ACTIVITIES** *(e.g., work, study, housework, family or leisure activities)* |  |
|  | I have no problems doing my regular activities | **[ ]** |
|  | I have slight problems doing my regular activities | **[ ]** |
|  | I have moderate problems doing my regular activities | **[ ]** |
|  | I have severe problems doing my regular activities | **[ ]** |
|  | I am unable to do my regular activities | **[ ]** |
|  |  |  |
| **HQ004** | **PAIN / DISCOMFORT** |  |
|  | I have no pain or discomfort | **[ ]** |
|  | I have slight pain or discomfort | **[ ]** |
|  | I have moderate pain or discomfort | **[ ]** |
|  | I have severe pain or discomfort | **[ ]** |
|  | I have extreme pain or discomfort | **[ ]** |
|  |  |  |
| **HQ005** | **WORRIES (ANXIETY) / DEPRESSION** |  |
|  | I am not worried or depressed | **[ ]** |
|  | I am slightly worried or depressed | **[ ]** |
|  | I am moderately worried or depressed | **[ ]** |
|  | I am severely worried or depressed | **[ ]** |
|  | I am extremely worried or depressed | **[ ]** |

10

0

20

30

40

50

60

80

70

90

100

5

15

25

35

45

55

75

65

85

95

| - We would like to know how good or bad your health is TODAY. |
| --- |
| - This scale is numbered from 0 to 100. |
| - 100 means the best health you can imagine. 0 means the worst health you can imagine. |
| - Mark an X on the scale to indicate how your health is TODAY. |
| - Now, please write the number you marked on the scale in the  box below. |

|  |
| --- |

**YOUR HEALTH TODAY =**
