# Supplementary material for: Health-related quality of life among adults living with chronic non-communicable diseases in the Ho Municipality of Ghana: a health facility-based cross-sectional study
Source: BMC Public Health. 2024 Mar 6;24:725. doi: 10.1186/s12889-024-18143-3 (PMC10918919; doi:10.1186/s12889-024-18143-3)
Supplement: Supplementary file 3 — Supplementary Material 3 [file 12889_2024_18143_MOESM3_ESM.docx]

**Additional File 3 – Measurement of Explanatory Variables**

| **Variable** | **Measurement** |
| --- | --- |
| Age | Self-reported age in years categorized as 30 – 39, 40 – 49. 50 – 59, and 60+. |
| Sex | Self-reported gender as male or female |
| Marital status | Self-reported marital status as never married, married, divorced/separated, or widowed |
| Education | Self-reported highest level of education completed as no formal education, primary, JHS/JSS/Middle School, SHS/SSS/O-Level or tertiary. |
| Religion | Self-reported religious affiliation as Christianity, Islam, or African Traditional Religion. |
| Ethnicity | Self-reported ethnic affiliation as Akan, Ewe, Guan or Ga-Dangme. |
| Diagnosed CNCD | Self-reported diagnosis of a chronic non-communicable disease as cancer, Chronic kidney disease, diabetes, hypertension or stroke. |
| Diagnosis duration | Self-reported length of time in years since diagnosis as <1, 1 – 5, 6 – 10, or 10+. |
| Comorbidity status | Self-reported presence or absence of other health conditions |
| Specific comorbidities | Self-reported list of other health conditions |
| Recommended behavioural/lifestyle changes | Self-reported adherence to recommended behavioural/lifestyle changes, including physical activity, dietary changes, smoke cessation, and alcohol intake moderation |
